# *In silico* Identification of Disrupted Myocardial Calcium Homeostasis as Proarrhythmic Trigger in Arrhythmogenic Cardiomyopathy

**DOI:** 10.3389/fphys.2021.732573

**Published:** 2021-09-24

**Authors:** Aurore Lyon, Chantal J. M. van Opbergen, Mario Delmar, Jordi Heijman, Toon A. B. van Veen

**Affiliations:** ^1^Division of Heart and Lungs, Department of Medical Physiology, University Medical Center Utrecht, Utrecht, Netherlands; ^2^The Leon Charney Division of Cardiology, New York University Grossmann School of Medicine, New York, NY, United States; ^3^Department of Cardiology, Cardiovascular Research Institute Maastricht, Maastricht University, Maastricht, Netherlands

**Keywords:** arrhythmogenic cardiomyopathy (ACM), plakophilin-2, computational modeling, calcium handling, arrhythmia

## Abstract

**Background:** Patients with arrhythmogenic cardiomyopathy may suffer from lethal ventricular arrhythmias. Arrhythmogenic cardiomyopathy is predominantly triggered by mutations in plakophilin-2, a key component of cell-to-cell adhesion and calcium cycling regulation in cardiomyocytes. Calcium dysregulation due to plakophilin-2 mutations may lead to arrhythmias but the underlying pro-arrhythmic mechanisms remain unclear.

**Aim:** To unravel the mechanisms by which calcium-handling abnormalities in plakophilin-2 loss-of-function may contribute to proarrhythmic events in arrhythmogenic cardiomyopathy.

**Methods:** We adapted a computer model of mouse ventricular electrophysiology using recent experimental calcium-handling data from plakophilin-2 conditional knock-out (PKP2-cKO) mice. We simulated individual effects of beta-adrenergic stimulation, modifications in connexin43-mediated calcium entry, sodium-calcium exchanger (NCX) activity and ryanodine-receptor 2 (RyR2) calcium affinity on cellular electrophysiology and occurrence of arrhythmogenic events (delayed-afterdepolarizations). A population-of-models approach was used to investigate the generalizability of our findings. Finally, we assessed the potential translation of proposed mechanisms to humans, using a human ventricular cardiomyocyte computational model.

**Results:** The model robustly reproduced the experimental calcium-handling changes in PKP2-cKO cardiomyocytes: an increased calcium transient amplitude (562 vs. 383 nM), increased diastolic calcium (120 vs. 91 nM), reduced L-type calcium current (15.0 vs. 21.4 pA/pF) and an increased free SR calcium (0.69 vs. 0.50 mM). Under beta-adrenergic stimulation, PKP2-cKO models from the population of models (*n* = 61) showed a higher susceptibility to delayed-afterdepolarizations compared to control (41 vs. 3.3%). Increased connexin43-mediated calcium entry further elevated the number of delayed-afterdepolarizations (78.7%, 2.5-fold increase in background calcium influx). Elevated diastolic cleft calcium appeared responsible for the increased RyR2-mediated calcium leak, promoting delayed-afterdepolarizations occurrence. A reduction in RyR2 calcium affinity prevented delayed-afterdepolarizations in PKP2-cKO models (24.6 vs. 41%). An additional increase in I_NCX_ strongly reduced delayed-afterdepolarizations occurrence, by lowering diastolic cleft calcium levels. The human model showed similar outcomes, suggesting a potential translational value of these findings.

**Conclusion:** Beta-adrenergic stimulation and connexin43-mediated calcium entry upon loss of plakophilin-2 function contribute to generation of delayed-afterdepolarizations. RyR2 and NCX dysregulation play a key role in modulating these proarrhythmic events. This work provides insights into potential future antiarrhythmic strategies in arrhythmogenic cardiomyopathy due to plakophilin-2 loss-of-function.

## Introduction

Arrhythmogenic cardiomyopathy (ACM) is an inherited cardiac disease characterized by fibrofatty replacement of the cardiac muscle, predominantly in the right ventricle. Individuals with ACM suffer from an increased risk of ventricular arrhythmias and sudden cardiac death (SCD), often occurring in young adults during exercise, in early (asymptomatic) stages of the disease (Corrado et al., 2017). However, disease penetrance is incomplete, and imaging techniques are at present unable to detect subclinical stages of the disease (Philips and Cheng, 2016), making early detection of ACM to prevent SCD challenging (Groeneweg et al., 2015). A better understanding of the mechanisms underlying ACM is needed to identify markers of early disease and predict potential arrhythmic events.

ACM can be caused by mutations in genes coding for desmosomal proteins. Among these, one of the most affected genes is *PKP2*, coding for the protein plakophilin-2 (PKP2). PKP2 is a desmosomal protein present in the intercalated disks of cardiac cells. As such, it plays a role in cell to cell adhesion, but as a component of the connexome, it also influences various molecular pathways. A dysfunction of these mechanisms would disrupt transcriptional events in the cardiomyocyte. The consequences of PKP2 deficiency in the cardiomyocyte remain poorly known, but recent studies have shown the crucial role of PKP2 in the translation of signals originating at the cell junction into intracellular signals controlling structural and electrical cardiomyocyte components, especially connexin43 (Oxford et al., 2007), voltage-gated sodium channel (Sato et al., 2009), and calcium cycling (Cerrone et al., 2017; Austin et al., 2019). This influence on calcium homeostasis suggests a key role for PKP2 loss-of-function in arrhythmogenicity, even in absence of structural disease (Cerrone et al., 2017).

A cardiomyocyte-specific PKP2 conditional knockout (PKP2-cKO) mouse model has previously been used to study the role of PKP2 in cardiomyocyte pathophysiology, and seemed to replicate critical components of ACM disease onset and progression in humans (Cerrone et al., 2017). This model can therefore serve as helpful tool to investigate early cellular events that trigger arrhythmia upon loss of PKP2 function. Among other things, these studies have revealed that PKP2 loss-of-function promotes disruption of intracellular calcium handling, resulting in an increased susceptibility toward arrhythmias induced by beta adrenergic stimulation (Cerrone et al., 2017; van Opbergen et al., 2019). Important proarrhythmic modifications include increased permeability of connexin43 (Cx43) hemichannels for calcium ions, potentially because of a weakened intercellular adhesion, leading to an increased calcium influx into cardiomyocytes (Kim et al., 2019). In addition, a reduced expression of the ryanodine receptor (RyR2), in combination with an increased calcium sensitivity of the channel, has been reported in PKP2-cKO hearts (Kim et al., 2019). RyR2 is an important regulator of sarcoplasmic reticulum (SR) calcium release and its activity is modulated by the cytoplasmic calcium concentration (Eisner et al., 2004). Therefore, RyR2 dysfunction predisposes to spontaneous SR calcium release events, playing a key role in arrhythmogenicity (Kim et al., 2019). However, the relative contribution of these calcium-handling alterations (Cx43-permeability and RyR2 calcium leak) to arrhythmia initiation, as well as the modulatory role of BARS in this model remain incompletely understood.

Computational models provide a controlled environment to evaluate the influence of distinct parameters on cellular electrophysiology and thereby may be very helpful to uncover mechanisms contributing to the arrhythmogenicity of PKP2-cKO cardiomyocytes. Computational models have shown to be effective at providing mechanistic insights into the dysregulation of cardiomyocyte calcium-handling (Sutanto et al., 2020). Previous studies have used computer models to demonstrate the role of aberrant sodium-current kinetics in facilitating reentry-based arrhythmias upon reduced presence of PKP2 (Deo et al., 2011), or to confirm the experimentally observed calcium-handling abnormalities (Cerrone et al., 2017). However, none of these studies focused on the underlying mechanisms by which calcium-handling abnormalities due to PKP2 loss-of-function may lead to arrhythmias.

In this paper, we adapted a computer model of mouse cardiac electrophysiology (Morotti et al., 2014), based on recent experimental data, to better understand how BARS and increased calcium influx through Cx43 hemichannels may contribute to proarrhythmic events in PKP2-cKO cardiomyocytes. Using a population-of-models approach (Britton et al., 2013), we show the synergistic effects of BARS and Cx43-mediated calcium entry in the generation of proarrhythmic delayed-afterdepolarizations (DADs), as well as the role of RyR2 and sodium-calcium exchanger (NCX) dysregulation in modulating these events. In PKP2-cKO cardiomyocytes, an increased diastolic cleft calcium was responsible for an increased RyR2-mediated calcium leak under BARS, leading to the occurrence of DADs. Reducing the affinity of RyR2 for calcium prevented the occurrence of DADs. In addition, increasing NCX activity further reduced DAD occurrence by lowering diastolic cleft calcium levels. Similar results were obtained in a human ventricular cardiomyocyte model. These cellular data provide initial insights into potential future antiarrhythmic strategies in ACM due to loss-of-function of PKP2.

## Materials and Methods

**Figure 1:**
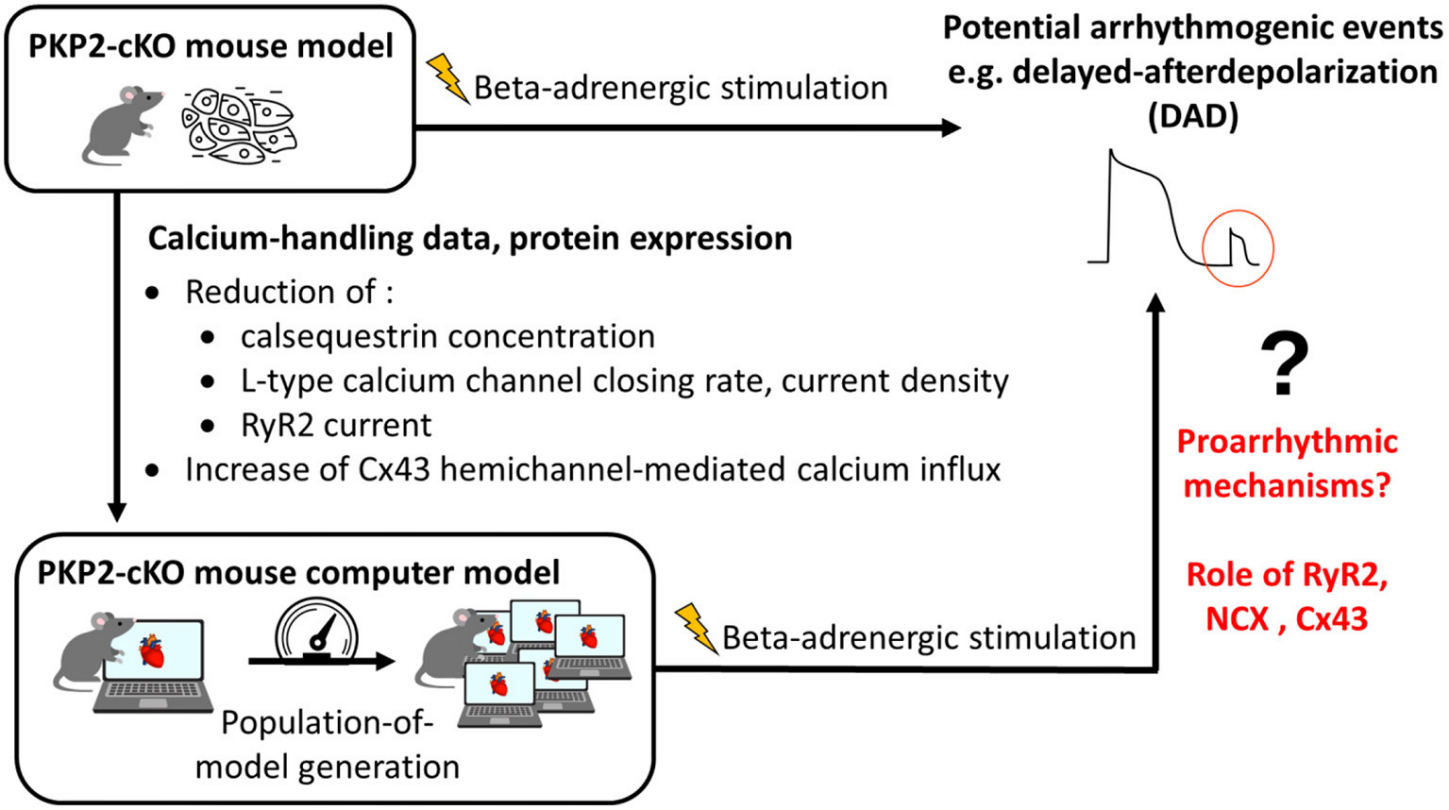
Methodology of combining experimental data with computational modeling to understand proarrhythmic mechanisms due to loss of plakophilin-2 (PKP2). PKP2-cKO, PKP2 conditional knock-out; RyR2, ryanodine receptors 2; Cx43, connexin43; NCX, sodium-calcium exchanger.

The overall methodology is illustrated in Figure 1. Details are provided in the subsections below.

### Experimental Calcium-Handling Data: PKP2 Conditional Knock-Out Mouse Model

Calcium imaging and patch-clamp data were obtained from previously published studies using a cardiomyocyte-specific, tamoxifen-activated, PKP2-cKO mouse model (Cerrone et al., 2017; Kim et al., 2019). Published differences in levels of proteins involved in calcium-handling, action potentials (AP) characteristics and calcium-transient properties were incorporated. In addition, the arrhythmia susceptibility upon an isoproterenol challenge was evaluated.

### PKP2-cKO Computer Simulations

Computer simulations of cardiomyocyte electrophysiology were performed using a validated state-of-the-art mouse ventricular cardiomyocyte model by Morotti et al. (2014), which also incorporates BARS signaling. Experimental characteristics from PKP2-cKO mouse cardiomyocytes were modeled as described below, following the mathematical simulations presented in Cerrone et al. (2017). Calsequestrin concentration was reduced to 45.3% of its original value. The closing rate of the L-type calcium channels was reduced to 75% of its original value and the current density of the L-type calcium current to 50% of its original value. Maximal calcium flux through the RyR2 and junctional volume were adjusted to 60% of their original value to account for RyR2 decreased expression. Model changes are summarized in Table 1.

**Table 1:** Summary table of the parameters changed in the original model (Morotti et al., 2014) to simulate the PKP2-cKO experimental characteristics, their interpretation, their values in the original WT and PKP2-cKO model and the relative change between WT and PKP2-cKO.

**Model parameter**	**Interpretation**	**WT value**	**PKP2-cKO value**	**Relative change**
Vjunc	Junctional volume	1.78e-14 L	1.07e-14 L	×60%
Bmax_Csqn	Calsequestrin buffer concentration	2.7 mM	1.22 mM	×45.3%
ICa_scale	Scaling of I_Ca, L_	1	0.5	×50%
r2m2	L-type calcium channel (LTCC) closing rate	0.38 ms^−1^	0.28 ms^−1^	×75%
ks	SR Ca release rate	25 ms^−1^	15 ms^−1^	×60%
kleak	SR Ca passive leak rate	1.22.10^−05^ ms^−1^	7.32. 10^−06^ ms^−1^	×60%

The effect of increased calcium influx via Cx43 hemichannels (reported in Cerrone et al., 2017; Kim et al., 2019) was assessed by varying the maximum conductance of the background calcium current (I_CaB_). In the simulations presented here, PKP2-cKO Cx43 hemichannel-mediated calcium entry is modeled by a 1.5-fold increase in I_CaB_ (as reported in Kim et al., 2019). To assess the potential impact of this influx on electrophysiological properties, simulations without I_CaB_ increase as well as with a larger Cx43 hemichannel-mediated calcium entry (simulated as a 2.5-fold increase in I_CaB_) were also performed. In addition, the effect of BARS on electrophysiological properties of PKP2 cardiomyocytes was modeled as described in Morotti et al. (2014).

Models were paced at 1 Hz for 500 s and AP and calcium-handling properties were recorded. The presence of DADs was quantified as secondary calcium transients with an amplitude > 100 nM after *t* = 400 ms. The model was implemented in MATLAB and solved using ODE15s and is available for download at https://github.com/aurora2093.

### Population-of-Models Approach

To evaluate the generalizability of the findings from the single model, take into account the effect of inter-subject variability on the electrophysiological properties modeled in this study, and quantify the relative contribution of modulating factors involved in DAD generation, we used a population-of-models approach (Britton et al., 2013; Muszkiewicz et al., 2016). The maximum conductances of eight major ionic currents (I_Na_, I_NaL_, I_CaL_, I_Kr_, I_K1_, I_to_, I_NCX_, I_NaK_) and the activity of RyR2 and SERCA2a were scaled using Latin-hypercube sampling as previously described (Britton et al., 2013; Ledezma et al., 2018). Five-hundred models were generated, and this control population was calibrated based on AP properties. Models were considered physiological if AP duration was between 20 and 80 ms, peak membrane potential larger than 25 mV, resting membrane potential (RMP) lower than −75 mV, upstroke duration lower than 10 ms and if the membrane potential was lower than RMP + 2 mV after 400 ms. Non-physiological models were rejected. In total, 61 out of 500 models that met these criteria were included in the analysis and the PKP2 remodeling as presented in section PKP2-cKO Computer Simulations was applied to these models.

### Human Model

To assess the translatability of our findings to human cellular electrophysiology, we simulated, as a proof-of-principle, the effects of the PKP2-cKO alterations detailed in section PKP2-cKO Computer Simulations in a human ventricular cardiomyocyte model. We adapted the Grandi et al. model (Grandi et al., 2010) which exhibits a similar structure as the Morotti et al. mouse model (especially in terms of RyR2 description). BARS and Cx43-mediated calcium influx were modeled as in section PKP2-cKO Computer Simulations.

### Statistical Analysis

Continuous variables with normal distribution are expressed as mean ± standard deviation. Categorical variables are presented as observed number with percentage. Normally distributed data were compared using *t*-tests. Non-normally distributed data were compared using the Mann–Whitney U-test. Statistical significance was assumed when *p* < 0.05. Statistical tests were performed using MATLAB.

## Results

### The Computer Model Recapitulates Experimentally-Measured Calcium-Handling Properties

**Figure 2:**
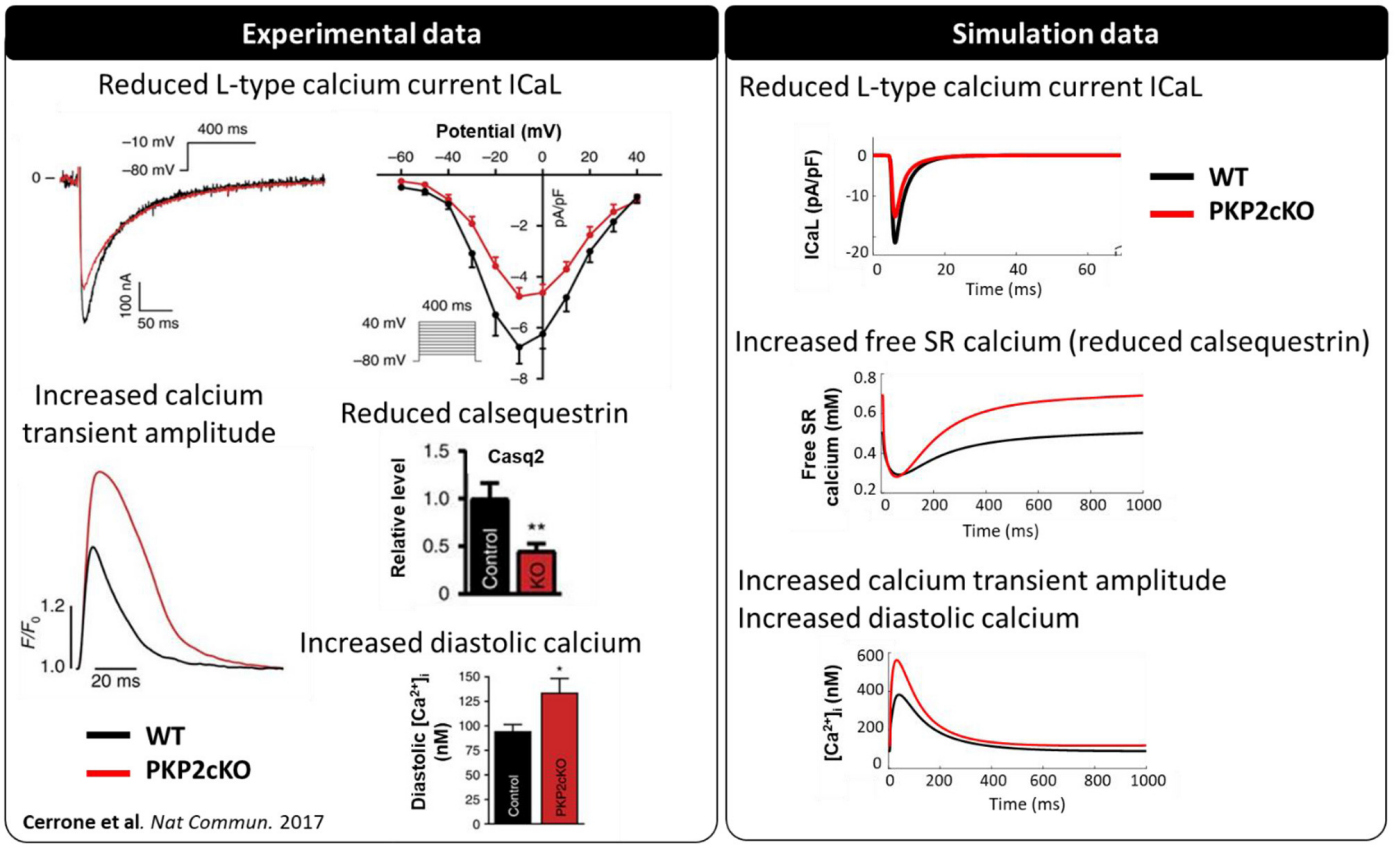
Calcium-handling properties [L-type calcium current (I_Ca,L_), calsequestrin protein levels, calcium transient and diastolic calcium concentrations] measured experimentally (modified from Cerrone et al., 2017) (^**^p < 0.01) in wildtype (WT) (black) and PKP2-cKO (red) cardiomyocytes (left), and corresponding simulated data [I_Ca,L_, free sarcoplasmic reticulum (SR) calcium concentration, calcium transient] (right).

**Figure 3:**
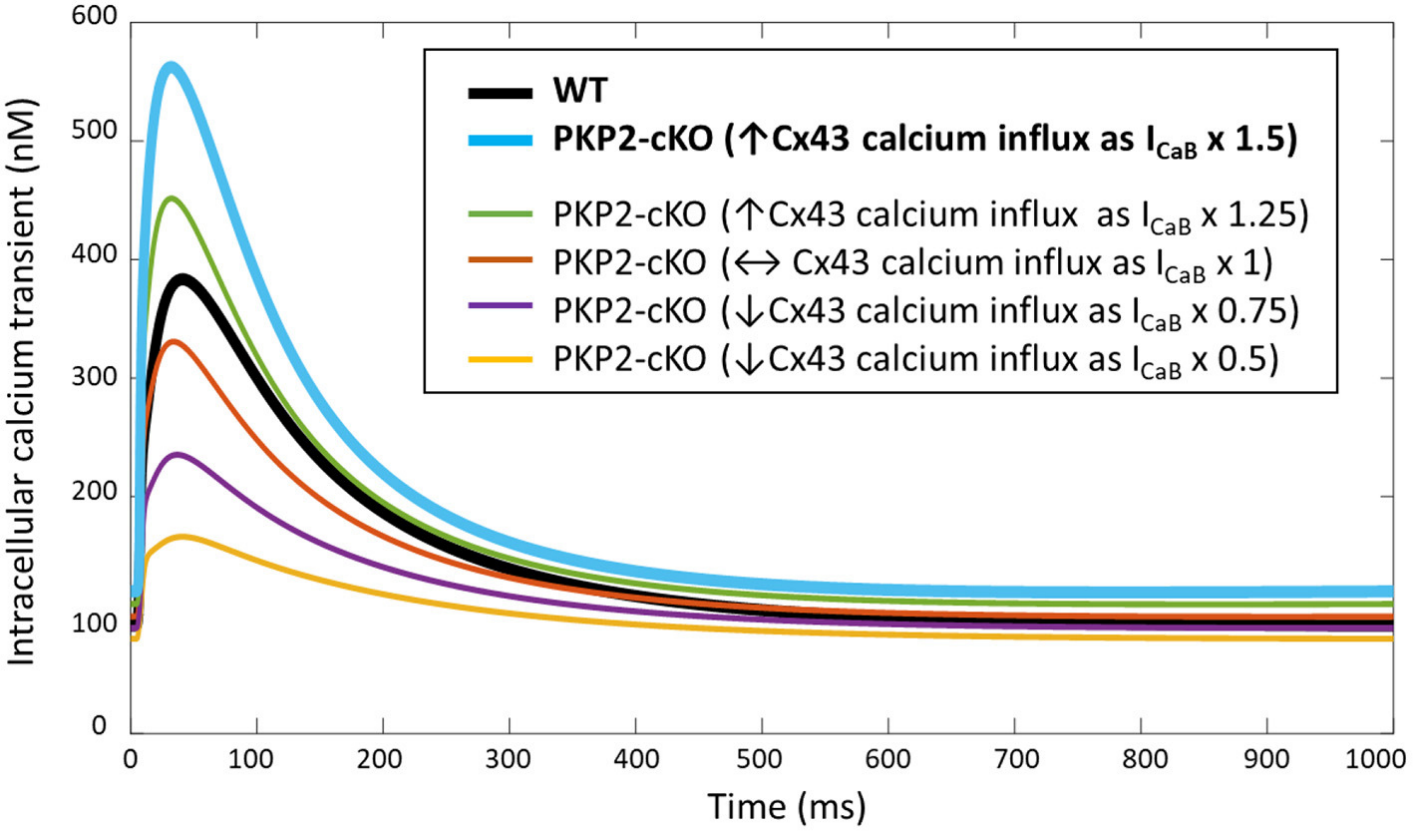
Intracellular calcium transient in WT and PKP2-cKO models with varying scaling of I_CaB_ (1.5 – baseline model, and 1, 0.5, 0.75, 1.25), to mimic different magnitudes of background calcium influx *via* Cx43 hemichannels.

PKP2-cKO changes were incorporated in the model as described in Methods. The model was able to recapitulate the experimentally observed changes in calcium-handling properties (Cerrone et al., 2017). The PKP2 model displayed an increased calcium transient amplitude compared to control (562 vs. 383 nM), as well as increased diastolic calcium levels (120 vs. 91 nM). Peak L-type calcium current was reduced (15.0 vs. 21.4 pA/pF) and the amount of free calcium in the SR was increased (0.69 vs. 0.50 mM), due to the reduced SR calcium buffering capacity by calsequestrin (Figure 2). Simulations revealed that the increased background calcium influx via Cx43 hemichannels (I_CaB_) contributed largely to the increased calcium transient amplitude, which was observed experimentally in PKP2-cKO cardiomyocytes (Figure 3).

### Beta-Adrenergic Stimulation and Cx43-Mediated Calcium Entry Contribute to DAD Occurrence

**Figure 4:**
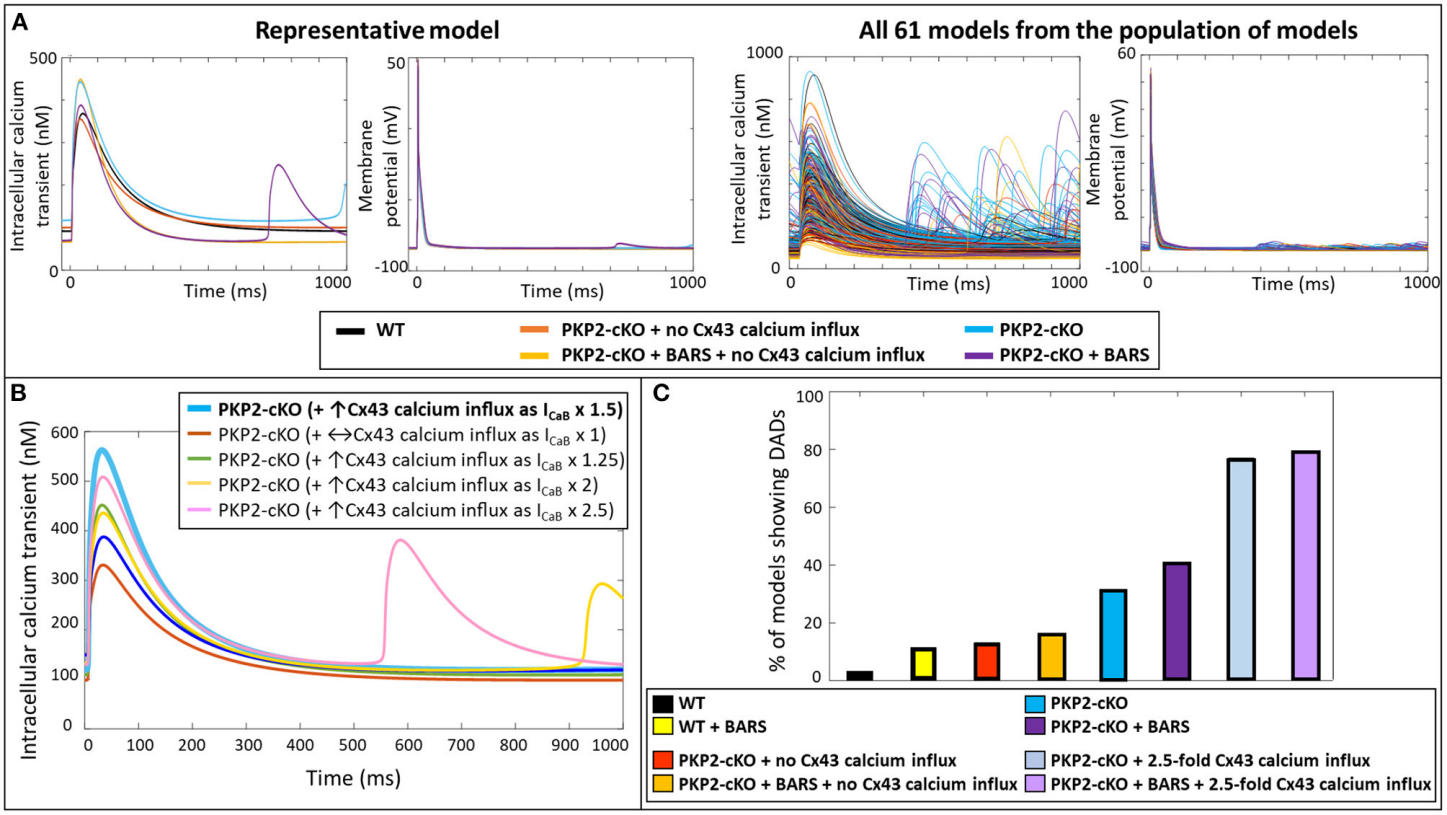
**(A)** Top: Representative calcium transient and membrane potential traces of the wildtype (WT) and PKP2-cKO model, with and without BARS and the PKP2-cKO model with no Cx43 hemichannels-mediated calcium influx, with and without BARS. Please note the occurrence of a DAD-like event in the presence of PKP2-cKO under BARS (purple line) but absence of these events without Cx43 hemichannels-mediated calcium influx. Bottom: Calcium transient and membrane potential traces for all models from the population of models in the five situations described above. **(B)** Calcium transient in PKP2-cKO models with various increments of Cx43 hemichannel-mediated calcium influx showing the occurrence of DADs from I_CaB_ x 2. **(C)** Percentage of models showing DADs under control (wildtype, WT) conditions with and without BARS, PKP2-cKO conditions with no Cx43 hemichannel-mediated calcium influx with and without BARS, PKP2-cKO conditions (1.5-fold increase in Cx43 hemichannel-mediated calcium influx) with and without BARS, and PKP2-cKO conditions in addition of an even larger increase in Cx43 hemichannel-mediated calcium influx (2.5-fold) with and without BARS.

Simulating BARS in the PKP2-cKO cell model without Cx43 calcium influx led to an increased calcium transient peak (448 vs. 355 nM), a reduction in calcium transient duration (550 vs. 681 ms) and a reduction in diastolic calcium levels (66 vs. 103 nM). In combination with an increased Cx43 calcium influx, BARS promoted spontaneous diastolic SR calcium-release events, which translated into the occurrence of DADs (occurring at *t* = 713 ms) (Figure 4A, top). Figure 4A (bottom) shows the calcium transient and AP traces in all models from the population of models. Of note, a larger (2.5-fold instead of 1.5-fold) increase in I_CaB_ was sufficient to induce DADs in the PKP2-cKO model, even in the absence of BARS (Figure 4B).

Subsequently, we employed a population-of-models approach to assess the generalizability of our findings. This approach confirmed the synergistic effects of BARS and increased Cx43-mediated calcium entry on the occurrence of DADs. At baseline, DADs were observed in 3.3% (*n* = 2) of control models and 13.1% (*n* = 8) of PKP2-cKO models without Cx43 calcium influx. Under BARS, 11.4% (*n* = 7) of controls and 16.4% (*n* = 10) of the PKP2-cKO models without Cx43 calcium influx showed DADs. Upon a 1.5-fold increased Cx43 hemichannel mediated calcium influx, 31.2% (*n* = 19) of the PKP2-cKO models showed DADs, vs. 41% (*n* = 25) under BARS (Figure 4C). With an even larger increase in calcium influx (2.5-fold), the contribution of BARS to the occurrence of DADs became smaller (77.1%, *n* = 47 without BARS vs. 78.7%, *n* = 48 with BARS).

### Ryanodine Receptor Dysfunction Contributes to the Occurrence of DADs

**Figure 5:**
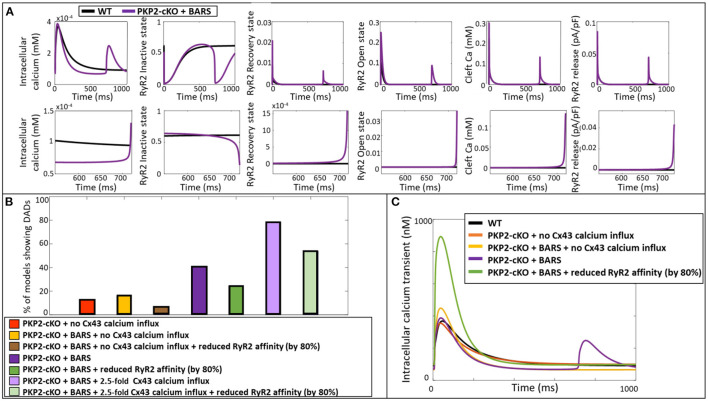
**(A)** Representation of the intracellular calcium transient, RyR2 configuration (inactive, recovery and open state), cleft calcium levels and RyR2-mediated calcium release in the wildtype model (blue) and PKP2-cKO model with BARS (1.5-fold Cx43 hemichannel-mediated calcium influx incorporated) (purple). Bottom: Zoom-in on the zone of DAD occurrence. **(B)** Percentage of models showing DAD-like events when comparing PKP2-cKO model with no Cx43 hemichannel-mediated calcium influx with and without BARS, with or without reduced RyR2 affinity, PKP2-cKO model plus BARS (1.5-fold Cx43 hemichannel-mediated calcium influx incorporated), with or without reduced RyR2 affinity, as well as the PKP2-cKO model plus BARS and 2.5-fold Cx43 hemichannel-mediated calcium influx, with or without reduced RyR2 affinity. **(C)** Representative calcium transient traces of the different models. Please note the disappearance of DADs when reducing the RyR2 calcium affinity in the PKP2-cKO model in presence BARS (1.5-fold Cx43 hemichannel-mediated calcium influx still incorporated).

Using the ability of computer modeling to investigate the contribution of individual parameters in the model, we compared the specific configurational changes in RyR2 states (inactive, open, recovered) at the onset of the DAD between control and PKP2-cKO with BARS. The RyR2 open probability was increased in the PKP2-cKO model compared to control, leading to an increased RyR2-mediated SR calcium leak. This resulted in increased cleft calcium levels, which in turn promoted further RyR2 opening, spontaneous SR calcium release events and the appearance of the DADs (Figure 5A).

To confirm the causal link between RyR2 hyperactivity and PKP2-cKO-related calcium-handling abnormalities, we reduced the RyR2 calcium affinity by 80% in our population of models, thereby lowering the sensitivity of RyR2 channels to increased cleft calcium concentrations. This reduction led to a decrease in the number of models that showed DADs (Figure 5B). This was effective in PKP2-cKO models with BARS without Cx43 calcium influx (6.6%, *n* = 4 vs. 16.4%, *n* = 10), in the PKP2-cKO models with BARS (1.5-fold increased Cx43 hemichannel-mediated calcium influx) (24.6%, *n* = 15 vs. 41%, *n* = 25), as well as in the PKP2-cKO models with BARS plus an even larger (2.5-fold) increase in Cx43 hemichannel-mediated calcium influx (54.1%, *n* = 33 vs. 78.7%, *n* = 48). Figure 5C shows a representative example of the effect of reducing RyR2 calcium affinity on calcium transient characteristics, clearly preventing DADs.

### Reduced NCX Activity Promotes DADs by an Increase in Diastolic Cleft Calcium

**Figure 6:**
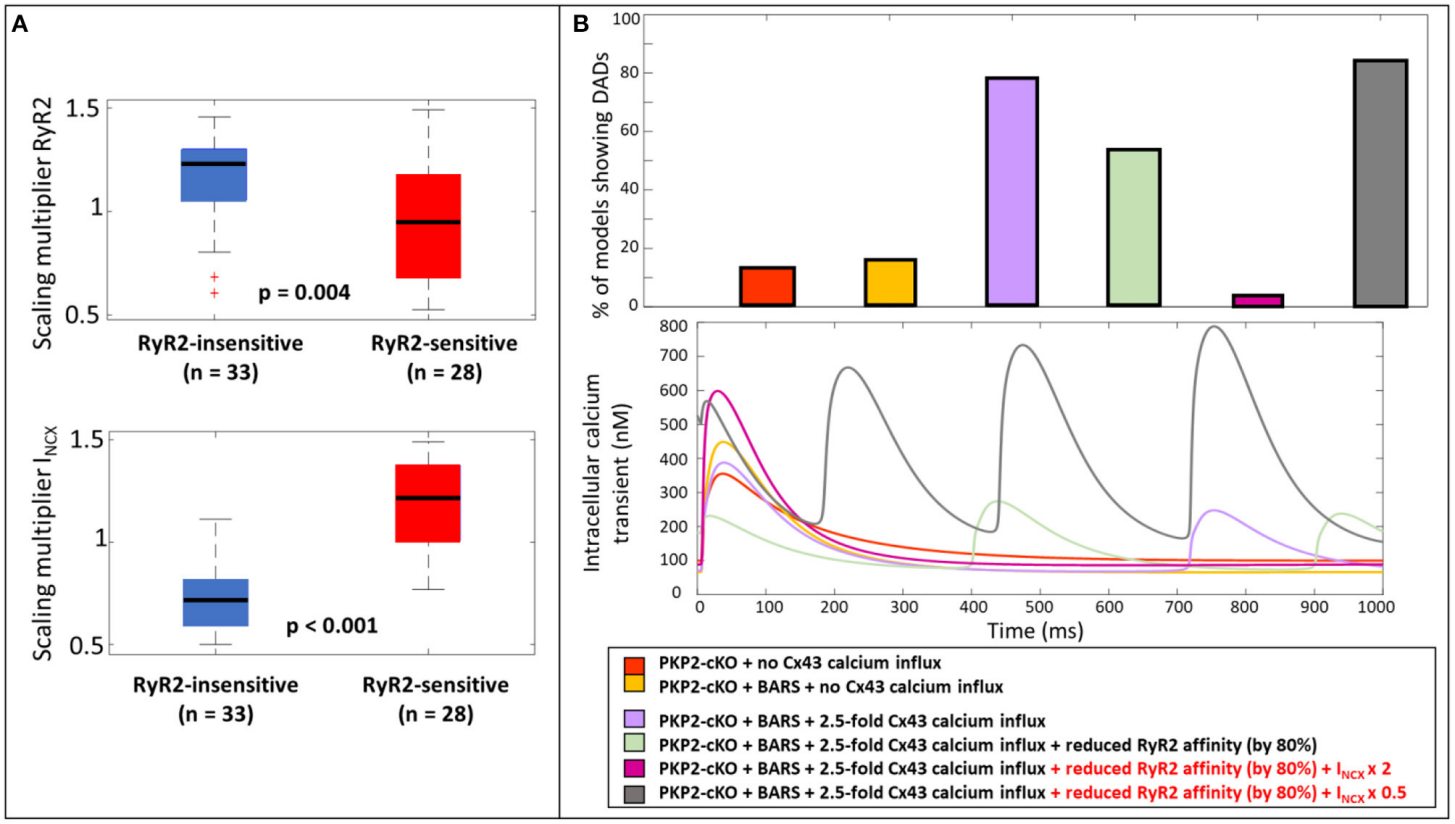
**(A)** Scaling multiplier of RyR2 and I_NCX_ between models that still show DADs after a reduction in RyR2 calcium affinity (RyR2-insensitive, blue) and the ones who do not show DADs after reduction (RyR2-sensitive, red). **(B)** Percentage of models showing DADs when comparing; the PKP2-cKO model with no Cx43 hemichannel-mediated calcium influx, PKP2-cKO model plus BARS (1.5-fold Cx43 hemichannel-mediated calcium influx incorporated), with or without reduced RyR2 affinity, the PKP2-cKO model plus BARS (1.5-fold Cx43 hemichannel-mediated calcium influx), with or without reduced RyR2 activity, doubled I_NCX_ in the RyR2-insensitive group or a 50% reduction of I_NCX_ in the RyR2-sensitive group (top); the corresponding calcium traces (bottom).

To further understand why the reduction in RyR2 calcium affinity did not remove the occurrence of DADs in all models with elevated Cx43 hemichannel-mediated calcium influx (2.5-fold) (54.1%, *n* = 33, Figure 5B), we analyzed the parameters that varied during the construction of the population of models. We compared the models in which reducing RyR2 calcium affinity removed the occurrence of DADs (RyR2-sensitive), with the models that still exhibited a DAD after a reduction in RyR2 calcium affinity (RyR2-insensitive). Significant parameter differences were observed in the scaling multipliers of RyR2 (*p* = 0.004, Figure 6A) and I_NCX_ (*p* < 0.001, Figure 6A). RyR2-insensitive models had a lower I_NCX_ maximum conductance multiplier compared to the RyR2-sensitive models (0.72 ± 0.15 vs. 1.18 ± 0.21, *p* < 0.001), suggesting an additional and modulating role for NCX in development of spontaneous SR calcium release events.

To analyze the role of NCX in modulating the occurrence of spontaneous calcium releases and DADs in the PKP2-cKO model plus BARS (including 1.5 fold Cx43 hemichannel-mediated calcium influx), we doubled I_NCX_ in the models from the RyR2-insensitive group. This greatly reduced the number of models showing DADs; 3.3% (*n* = 2), upon doubled I_NCX_ vs. 54.1% (*n* = 33), with RyR2 calcium affinity reduction only). In line with this observation, a 50% reduction in I_NCX_ in RyR2-sensitive models greatly increased the occurrence of spontaneous calcium releases (83.6%, *n* = 51, Figure 6B).

**Figure 7:**
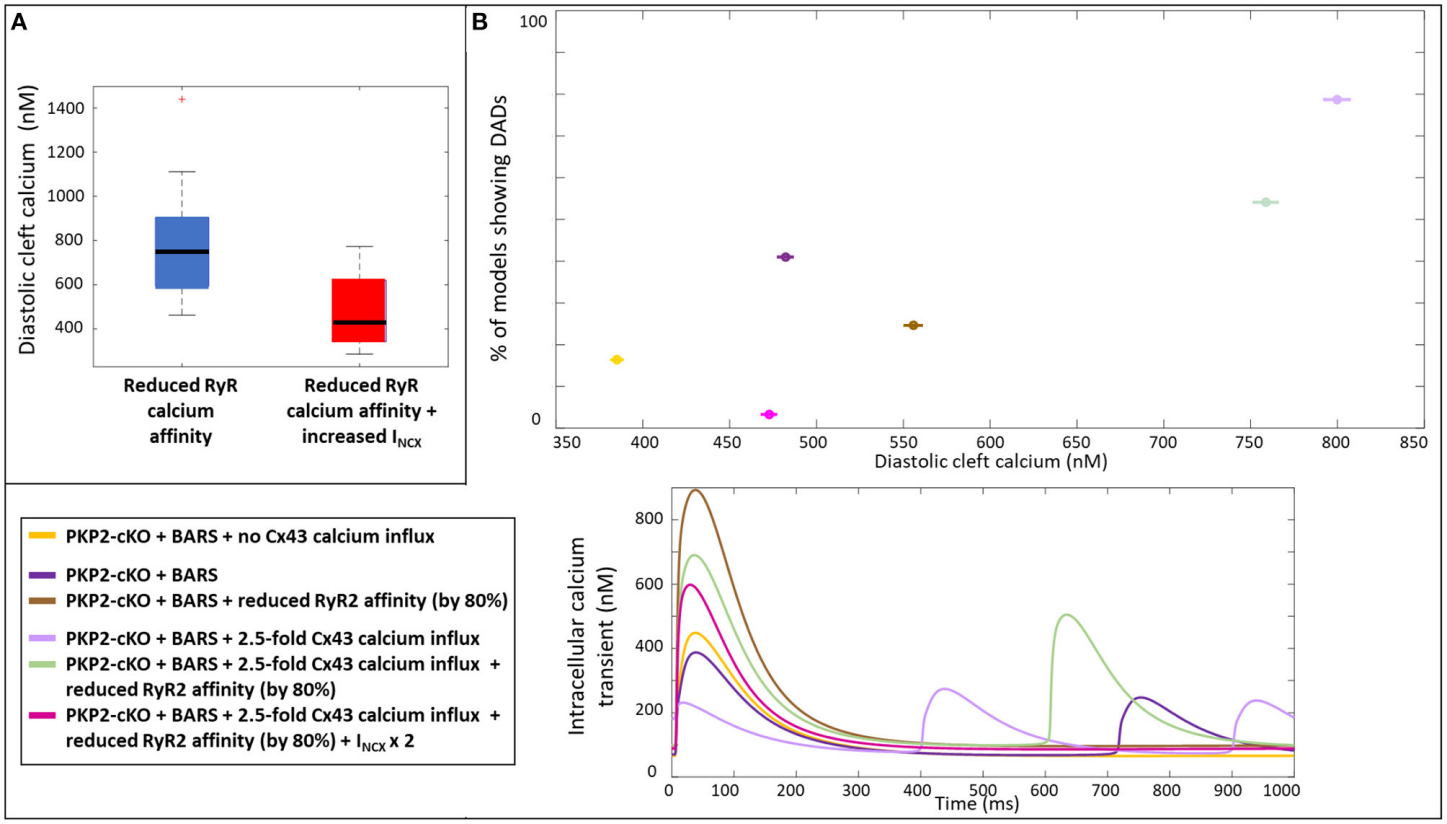
**(A)** Diastolic cleft calcium levels upon a reduced RyR2 calcium affinity (blue) and on top of that an increased I_NCX_ (red). **(B)** Percentage of models showing DADs, plotted against the diastolic cleft calcium levels presented for six different situations in the PKP2-cKO model (top); Corresponding calcium traces (bottom).

We analyzed the mechanisms by which an increase in I_NCX_ may help to reduce the occurrence of spontaneous calcium releases by comparing the diastolic cleft calcium concentration with and without an increase in I_NCX_. Diastolic cleft calcium level was much lower with increased I_NCX_ (471 ± 150 nM), compared to baseline I_NCX_ (758 ± 195 nM; Figure 7A), suggesting that an NCX-mediated lowering of diastolic cleft calcium level underlies the potential antiarrhythmic effects of increased NCX. In agreement, Figure 7B shows the relationship between diastolic calcium levels and likelihood of DADs in the various situations [PKP2-cKO with BARS without Cx43 calcium influx, PKP2-cKO with BARS (1.5-fold Cx43 hemichannel-mediated calcium influx), PKP2-cKO with BARS, 1.5-fold Cx43 calcium influx and reduced RyR2 calcium affinity, and PKP2-cKO with BARS, 1.5-fold calcium influx, reduced RyR2 calcium affinity and increased NCX]. This analysis revealed an increased occurrence of DADs with larger diastolic calcium levels (78.7% at 799 nM in PKP2-cKO plus BARS and 1.5-fold Cx43-mediated calcium influx vs. 3.3% at 473 nM in PKP2-cKO plus BARS, 1.5-fold calcium influx, reduced RyR2 calcium affinity and increased I_NCX_ in RyR2-insensitive models, correlation coefficient *r* = 0.861). A reduction in RyR2 calcium affinity and an increase in I_NCX_ apparently facilitate a reduction in diastolic cleft calcium level, thereby reducing the number of models showing DADs.

### Translation to Humans

**Figure 8:**
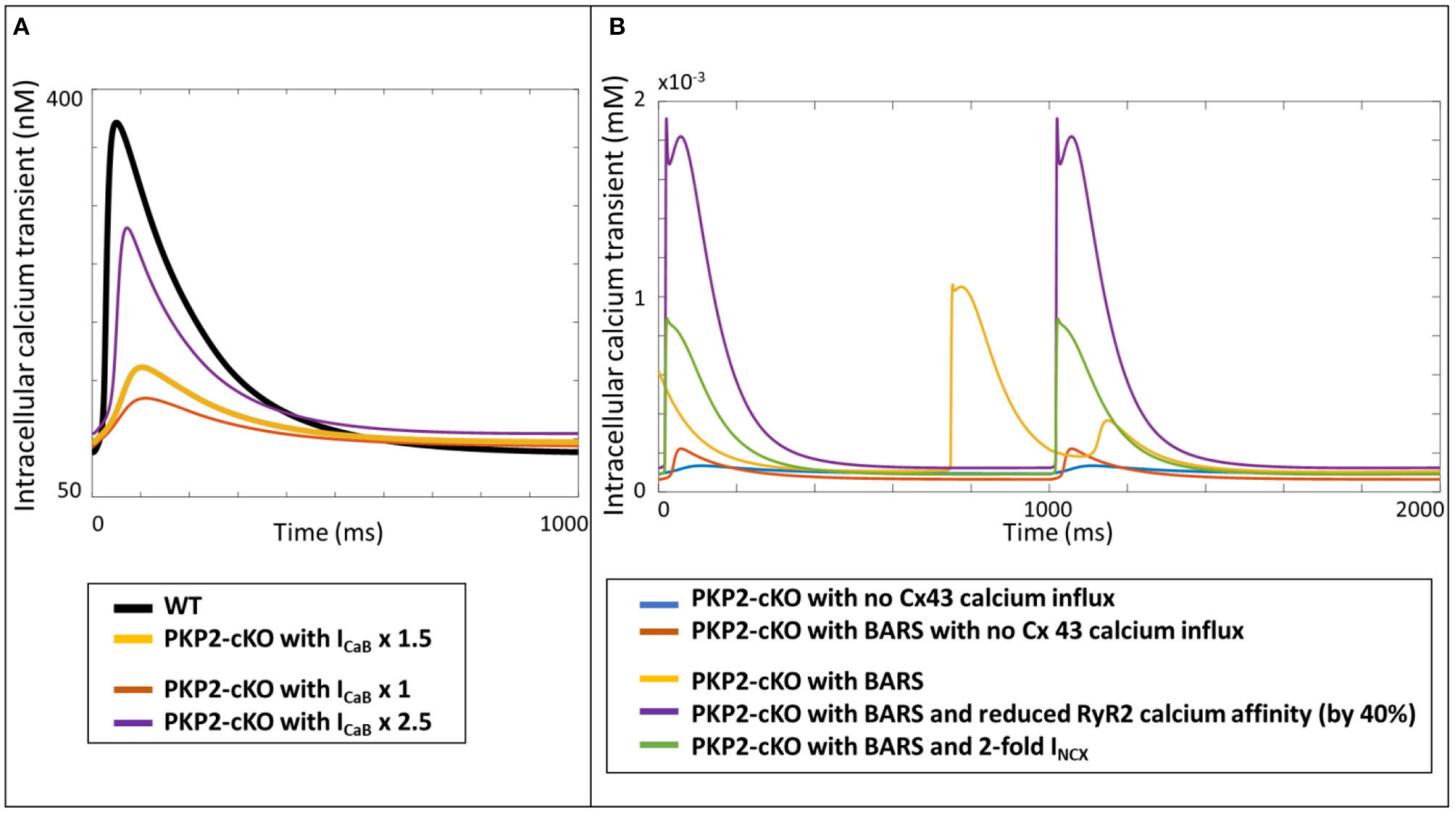
**(A)** Calcium transients in the human WT, PKP2-cKO and PKP2-cKO model, with various increments of Cx43 hemichannels-mediated calcium entry, applied in the Grandi human ventricular cardiomyocyte model. **(B)** Calcium transients in the PKP2-cKO model with no Cx43 calcium influx with and without BARS, and PKP2-cKO model plus BARS, RyR2 calcium affinity reduction and increased I_NCX_.

As a proof-of-principle for the translational relevance of our computational findings, based on experimental mouse data, for human ACM pathophysiology, we incorporated the measured PKP2-cKO changes in a human ventricular cardiomyocyte computer model. Increasing the Cx43 hemichannel-mediated calcium entry in WT (via an increase in I_CaB_) modulated the amplitude of the the calcium transient, like in the mouse. However, contrary to the mouse model, a 2.5-fold increase in I_CaB_ did not lead to an increased calcium transient amplitude in the human PKP2-cKO model, compared to control (Figure 8A). Under BARS and increased Cx43 hemichannel-mediated calcium entry (5-fold increase in I_CaB_), DADs occurred (yellow trace, Figure 8B). A reduction in RyR2 calcium affinity removed the DADs (purple trace) but increased the amplitude of the calcium transient considerably. An increase in NCX activity (2-fold) also removed the DADs, as seen in the murine PKP2-cKO computational model.

## Discussion

Pathophysiological remodeling of the heart is a complex orchestration of molecular alterations which, together, gradually may deteriorate normal performance toward heart failure (HF). The magnitude and importance of different underlying molecular mechanisms often changes during the transition toward HF, which makes it extremely difficult to pinpoint the most relevant ones for cardiac deterioration. This also applies to pathological alterations in the cardiac calcium homeostasis that gradually compromise contractility, but also enhance the susceptibility to arrhythmias. Pathophysiological mechanisms are often studied using experimental models like (transgenic) mice or human induced pluripotent stem cells-derived cardiomyocytes. In previous studies we have used the PKP2-cKO mouse model to study the endophenotype of PKP2 and ACM disease progression. This work identified disturbed calcium homeostasis as a major contributor toward cardiac arrhythmias in the early stage of the disease, and disease progression in general. To better understand and specify the molecular events contributing to the disturbed calcium handling in these cardiomyocytes, we employed the computational approach described in the current study. Using a combination of mouse-specific computer modeling and experimental data obtained from the PKP2-cKO mouse model, our work highlights the contribution of BARS and Cx43 hemichannel-mediated calcium entry in the generation of proarrhythmic DADs in this mouse model. Our simulations uncovered RyR2 and NCX dysregulation as important modulators of these proarrhythmic events. In PKP2-cKO cardiomyocytes, an increased diastolic cleft calcium was responsible for an increased RyR2-mediated calcium leak, leading to the occurrence of DADs. Reducing the affinity of RyR2 for cytosolic calcium prevented the occurrence of DADs. In addition, a further increase in NCX activity reduced the likelihood of DADs, by lowering the Cx43 hemichannel-mediated elevation of diastolic cleft calcium level. These cellular data provide initial insights into potential future antiarrhythmic strategies in ACM, due to loss-of-function of PKP2.

### PKP2-cKO Cellular Changes Are Captured by the Computer Model and the Population-of-Models Approach Captures Cell Variability

Our computer model convincingly reproduced the cellular changes measured in the PKP2-cKO mouse model; a reduced L-type calcium current, increased SR calcium load, increased calcium transient amplitude and increased diastolic calcium levels. Our simulations suggested that Cx43 hemichannel-mediated calcium entry is a key modulator of calcium transient amplitude in PKP2-cKO computational models. An increased Cx43 hemichannel-mediated calcium entry upon loss-of-function of PKP2 in mice has been demonstrated in previous studies (Kim et al., 2019; van Opbergen et al., 2019) and is likely the result of a disrupted cell-to-cell adhesion and/or orphan Cx43 hemichannels, allowing influx of calcium into the cardiomyocytes.

Interestingly, the effects of this Cx43 hemichannel related calcium entry were different in the human model, likely due to inter-species differences in cellular electrophysiology (discussed in more detail in section Translation to Human). The population-of-models approach allowed us to assess the generalizability of our findings by generating 61 different models with slightly varying baseline electrophysiological properties. This helped us to uncover an additional and potential key role for NCX by comparing the electrophysiological properties of the models that did, and those that did not, respond to a RyR2 calcium affinity reduction, and linked that to the occurrence of DADs.

### BARS and Cx43 Hemichannels-Mediated Calcium Entry Contribute to Arrhythmogenicity by PKP2-cKO

BARS and Cx43 hemichannel-mediated calcium entry both contributed to arrhythmogenic events in the PKP2-cKO computational models. This was illustrated by the number of models showing DADs under the conditions of PKP2-cKO plus BARS with and without Cx43 hemichannel-mediated calcium entry. This is in agreement with an enhanced susceptibility to isoproterenol induced arrhythmias in PKP2-cKO hearts (Kim et al., 2019). Cx43-mediated calcium entry had a larger contribution to the number of DADs than BARS, with a smaller increase in DAD occurrence with BARS in the presence of large Cx43-mediated calcium entry (2.5-fold increase in I_CaB_). In agreement, a large (2-fold) increase in Cx43-mediated calcium entry was sufficient to induce DADs even in the absence of BARS. This is in line with the experimental data reported in Kim et al. (2019), showing a larger amount of early and delayed after-transients in PKP2-cKO cardiomyocytes, even in the absence of BARS. This role of Cx43 hemichannel-mediated calcium entry in PKP2-cKO arrhythmogenesis is also consistent with recent studies showing its pro-arrhythmic effect (van Opbergen et al., 2019), and has been reported in other conditions such as heart failure (Smet et al., 2021) and muscular dystrophy (Patrick Gonzalez et al., 2015). Importantly, blocking Cx43 hemichannels with GAP19 normalized intracellular calcium homeostasis and reduced the occurrence of calcium sparks in PKP2-cKO cardiomyocytes (Kim et al., 2019), confirming the proarrhythmic effects of Cx43-mediated calcium entry.

### RyR2 and NCX Dysfunction Modulate the Occurrence of DADs in PKP2-cKO Models by Increasing Diastolic Calcium Levels

In the simulations, DADs occurred as a direct consequence of RyR2 dysfunction. The RyR2 open probability was increased in the PKP2-cKO model compared to control, leading to an increased RyR2-mediated SR calcium leak. Resulting from that, the diastolic cleft calcium levels were increased, promoting further opening of RyR2 channels, spontaneous SR calcium release and the development of DADs. Our modeling confirmed that a reduction in the calcium affinity of RyR2 decreased the number of PKP2-cKO models showing DADs, even back to baseline values.

A reduction in RyR2 calcium affinity was not sufficient to remove all DADs when the magnitude of Cx43 hemichannel calcium entry was so large that DADs occurred without BARS. An increase in I_NCX_, however, reduced the diastolic cleft calcium levels and removed any remaining DADs. Our previous experimental studies did not discover changes in I_NCX_, NCX protein levels and NCX mRNA levels in PKP2-cKO hearts (Kim et al., 2019). However, the population-of-models approach uncovered that NCX dysfunction, in addition to electrophysiological remodeling upon loss-of-function of PKP2, may act as a potential additional proarrhythmogenic factor. In a more general setting of HF, both unchanged and enhanced activity have been reported (Flesch et al., 1996; Schwinger et al., 1999; Sipido et al., 2002). Interestingly, unchanged NCX activity was associated with diastolic dysfunction whereas diastolic function in the case of enhanced NCX activity remained normal (Hasenfuss et al., 1999). This suggests that an enhanced NCX activity may be a biological compensatory mechanism to temper calcium handling dysregulation in the heart (Louch et al., 2010), substantiating our computational findings in this regard.

In our simulations, the DADs observed in PKP2-cKO cells originated from increased diastolic calcium levels. Thus, reducing the RyR2 calcium affinity and increasing I_NCX_ helped lowering diastolic calcium and preventing the occurrence of DADs. Increasing I_NCX_ was particularly powerful in reducing diastolic calcium levels, even removing DADs in the models that did not respond to the RyR2 inhibition alone. This highlights the complementary effects of reducing both the cytosolic influx of calcium from the SR (RyR2 effect) and normalizing diastolic cleft calcium levels (NCX effect). Interestingly, no particular role of intracellular sodium was found in our simulations, reinforcing the influence of calcium handling disturbances in arrhythmogenicity induced by loss-of-function of PKP2.

### Translation to Human

In order to investigate the translational value of our findings in mouse models, to human pathophysiology of ACM, we introduced the PKP2-cKO alterations in our human computational electrophysiology model. These simulations suggested that the mechanisms presented in this study have potential translational value, in spite of differences between murine and human electrophysiology. We observed that in human the calcium amplitude remained lower in the PKP2-cKO model, compared to control, despite an increased Cx43 hemichannel-mediated calcium entry. This could be caused by a difference in the balance in transsarcolemmel (I_CaL_ and NCX) and SR (SERCA) calcium fluxes between mice and human. Reducing I_CaL_ had more impact on the calcium transient amplitude in the PKP2-cKO human model, because I_CaL_ has a more profound role in calcium entry in human cardiomyocytes when compared to that in mice (Bers, 2008). However, despite these inter-species differences, the simulations showed that DADs caused by BARS and Cx43 hemichannel-mediate calcium entry were suppressed by a reduction in RyR2 calcium affinity, similar to what was shown in the mouse models. This suggests that the mechanisms presented here potentially translate to humans, which could open doors for future clinical management of patients with ACM. These results also reinforce the potential of computer modeling in translating experimental findings to patients, thereby hopefully bridging the gap in knowledge regarding the molecular mechanisms involved in the pathogenesis of ACM in humans. The latter being obviously compromised by the fact that human cardiac specimen to study these molecular alterations are virtually absent, especially when it comes to patients in the concealed phase of the disease, who might be at high risk for major arrhythmic events and SCD.

### Clinical Impact

Here, we propose a role for RyR2 dysfunction and NCX modulation as potential targets for the prevention/treatment of arrhythmias resulting from loss of PKP2 function. We suggest that a reduction in the calcium affinity of RyR2 and an increase in NCX activity may help to reduce the occurrence of arrhythmogenic events. This can have implications for potential therapeutic strategies, for example drugs modulating RyR2 activity, such as ent-*verticilide* (Batiste et al., 2019). Interestingly, our simulations suggest an additional anti-arrhythmic role for NCX stimulators, which would inhibit the reverse-mode of NCX. This could be further investigated experimentally in the PKP2-cKO mice. This is in agreement with previous studies reporting a cardioprotective effect of NCX stimulatory drugs like flecainide, although flecainide also is known to modulate RyR2 and the sodium current (Watanabe, 2019). Flecainide was effective in suppressing arrhythmic events through direct modulation of I_NCX_ in Andersen-Tawil syndrome-induced pluripotent stem cells-derived cardiomyocytes (Kuroda et al., 2017). Interestingly, patients with ACM do respond positively to flecainide (Ermakov et al., 2017; Bouvier et al., 2018) and this is current subject of an ongoing clinical trial (Zareba, 2021). This highlights the potential effect of the drug on calcium handling disturbances and includes modulation of NCX as a potential antiarrhythmic mechanism. In addition, our simulations suggested that Cx43-mediated calcium entry may be a major proarrhythmic trigger. Correcting this increased calcium entry could therefore be an additional potential therapeutic strategy.

### Limitations and Future Directions

In the simulations, we mimicked Cx43 hemichannel-mediated calcium entry by an increase in background calcium current. This reproduced a general entry of calcium in the cell, but the localization of the channel, residing in the perimeter of the gap junctions, as well as the entry of other ions like sodium were not included in the model. Future work may focus on describing in more detail the action of Cx43 hemichannels to investigate the influence of potential other factors (dynamically regulating calcium entry) in the mechanisms proposed here.

In addition, a thorough analysis of the mechanisms in humans is beyond the scope of this study, but our simulations show a promising translation of the mechanisms we propose here to human. Future work will focus on investigating the role of BARS, Cx43-mediated calcium entry, RyR2 and NCX dysfunction in humans. Finally, these results provide insights in cellular proarrhythmic events in PKP2 loss-of-function, but arrhythmias are inherently multicellular phenomena and extrapolation across these spatial scales remains challenging and beyond the scope of the current study.

## Conclusions

Using computer modeling combined with experimental data from mouse models, we uncovered the contribution of BARS and Cx43 hemichannel-mediated calcium entry in generation of proarrhythmic DADs in the PKP2-cKO model. In these cardiomyocytes, an increased diastolic cleft calcium was responsible for increased RyR2-mediated calcium leak, leading to the occurrence of DADs. A reduction in RyR2 calcium affinity prevented the occurrence of DADs and an increased NCX activity further reduced DAD occurrence by lowering diastolic cleft calcium levels. By uncovering additional mechanisms underlying arrhythmogenicity in PKP2-cKO cardiomyocytes, this work provides potential insights into future antiarrhythmic strategies in ACM due to dysfunction of PKP2.

## Data Availability Statement

The datasets presented in this study can be found in online repositories. The names of the repository/repositories and accession number(s) can be found at: https://github.com/aurora2093/FrontiersinPhysiology2021_PKP2.

## Author Contributions

AL, JH, and TV conceived the study, performed the data analysis, and drafted the manuscript. AL performed the computational simulations. MD, CO, and TV generated the used experimental data. AL, CO, MD, JH, and TV critically revised the manuscript. All authors approved the final version.

## Funding

This work was supported by Netherlands Cardiovascular Research Initiative: an initiative with support of the Dutch Heart Foundation (CVON 2015-12 eDETECT. 2018-30 PREDICT2 to TV), by a grant from the UU-3R Stimulation Fund (to AL), by NIH grants RO1-HL134328, RO1-HL136179 and RO1-HL145911 (MD), a Transatlantic Network of Excellence from the Leducq Foundation (MD), the Wilton W. Webster Fellowship in Pediatric Electrophysiology from Heart Rhythm Society (CO) and by the Netherlands Organization for Scientific Research NWO/ZonMW Vidi 09150171910029 to JH.

## Conflict of Interest

The authors declare that the research was conducted in the absence of any commercial or financial relationships that could be construed as a potential conflict of interest.

## Publisher's Note

All claims expressed in this article are solely those of the authors and do not necessarily represent those of their affiliated organizations, or those of the publisher, the editors and the reviewers. Any product that may be evaluated in this article, or claim that may be made by its manufacturer, is not guaranteed or endorsed by the publisher.

## Abbreviations

ACM: arrhythmogenic cardiomyopathy
AP: action potential
APD: action potential duration
BARS: beta-adrenergic stimulation
Cx43: connexin43
DAD: delayed afterdepolarization
HF: heart failure
NCX: sodium-calcium exchanger
PKP2: plakophilin-2
PKP2-cKO: PKP2 conditional knockout
RMP: resting membrane potential
RyR2: ryanodine receptors 2
SCD: sudden cardiac death
SR: sarcoplasmic reticulum
WT: wildtype.
